# Aberrant dynamin 2-dependent Na^+^/H^+^ exchanger-1 trafficking contributes to cardiomyocyte apoptosis

**DOI:** 10.1111/jcmm.12086

**Published:** 2013-07-09

**Authors:** Jun Li, Liang Xu, Jiangchuan Ye, Xiang Li, Dasheng Zhang, Dandan Liang, Xinran Xu, Man Qi, Changming Li, Hong Zhang, Jing Wang, Yi Liu, Yuzhen Zhang, Zhaonian Zhou, Xingqun Liang, Jue Li, Luying Peng, Weidong Zhu, Yi-Han Chen

**Affiliations:** a Key Laboratory of Arrhythmias of the Ministry of Education of China, East Hospital, Tongji University School of MedicineShanghai, China; b Institute of Medical Genetics, Tongji UniversityShanghai, China; c Department of Cardiology East Hospital, Tongji University School of MedicineShanghai, China; d Laboratory of Hypoxic Cardiovascular Physiology Shanghai Institutes for Biological Sciences, Chinese Academy of SciencesShanghai, China; e Department of Pathology and Pathophysiology, Tongji University School of MedicineShanghai, China

**Keywords:** Na^+^/H^+^ exchanger 1, dynamin, trafficking, apoptosis, cardiomyocytes

## Abstract

Sarcolemmal Na^+^/H^+^ exchanger 1 (NHE1) activity is essential for the intracellular pH (pH_i_) homeostasis in cardiac myocytes. Emerging evidence indicates that sarcolemmal NHE1 dysfunction was closely related to cardiomyocyte death, but it remains unclear whether defective trafficking of NHE1 plays a role in the vital cellular signalling processes. Dynamin (DNM), a large guanosine triphosphatase (GTPase), is best known for its roles in membrane trafficking events. Herein, using co-immunoprecipitation, cell surface biotinylation and confocal microscopy techniques, we investigated the potential regulation on cardiac NHE1 activity by DNM. We identified that DNM2, a cardiac isoform of DNM, directly binds to NHE1. Overexpression of a wild-type DNM2 or a dominant-negative DNM2 mutant with defective GTPase activity in adult rat ventricular myocytes (ARVMs) facilitated or retarded the internalization of sarcolemmal NHE1, whereby reducing or increasing its activity respectively. Importantly, the increased NHE1 activity associated with DNM2 deficiency led to ARVMs apoptosis, as demonstrated by cell viability, terminal deoxynucleotidyl transferase–mediated dUTP nick-end labelling assay, Bcl-1/Bax expression and caspase-3 activity, which were effectively rescued by pharmacological inhibition of NHE1 with zoniporide. Thus, our results demonstrate that disruption of the DNM2-dependent retrograde trafficking of NHE1 contributes to cardiomyocyte apoptosis.

## Introduction

Na^+^/H^+^ exchangers (NHEs), a ubiquitously expressed family of integral membrane proteins, play important roles in the regulation of intracellular pH (pH_i_) *via* H^+^ extrusion driven by transmembrane Na^+^ gradient [1]. To date, 12 mammalian isoforms of NHE have been identified, although NHE1 is the primary acid extrusion system in cardiac cells [2]. Several studies have demonstrated that sarcolemmal NHE1 dysfunction was closely related to cardiomyocyte death under pathophysiological conditions such as ischaemia-reperfusion injury, ventricular hypertrophy and myocardial infarction, underlying the pathophysiological importance of the fine-tuned NHE1 activity [3–6].

NHE1 activity is dynamically changed in response to the fluctuation of pH_i_, and is also subject to the modulation by various extracellular stimuli, such as growth factors, hormones, integrin engagement [7–9], and the intracellular messengers such as protein kinase A, mitogen-activated protein kinase and Ca^2+^/calmodulin complex [10–12]. The enhancement of NHE1 activity in parallel with the increase in its membrane expression implicated the involvement of a protein trafficking process in the regulation of NHE1 activity [13]. However, the molecular mechanism underlying this process remains undefined.

Dynamin (DNM) is a superfamily of large guanosine triphosphatases (GTPase) that hydrolyse GTP to form GDP [14]. Mammals express three DNM isoforms, of which DNM2 is ubiquitously expressed; DNM1 and DNM3 are predominantly distributed in neurons and testis respectively [15]. DNM is best known for its roles in membrane-trafficking processes, for example, caveolae-mediated and clathrin-dependent endocytosis, trafficking from the trans-Golgi network and membrane invagination/fission [16–19]. A recent study reported the regulatory effects of DNM on sarcolemmal L-type calcium channel [20], suggesting the potential role of DNM in the cardiomyocyte membrane-related protein trafficking. In this study, we aimed to determine whether cardiac DNM can modulate the trafficking and activity of sarcolemmal NHE1, and if so, to explore the related physiological implication.

## Materials and methods

### Construction of adenoviral vectors

The wild-type (WT) and the mutated form (K44A, defective in GTP binding) of DNM2 were used in the manipulation of DNM2 activity. Briefly, the cDNAs of rat DNM2^WT^/DNM2^K44A^ (obtained from ATCC; DNM2^WT^: MBA-94, DNM2^K44A^: MBA-95; http://www.atcc.org) were cloned into the pDONR221 vector using BP Clonase® enzyme mix (Invitrogen, Grand Island, NY, USA). The pDONR221 vectors containing the DNM2^WT^/DNM2^K44A^ sequences were subsequently recombined with the pAd/CMV/V5-DEST vector using LR Clonase® enzyme mix (Invitrogen) to yield the pAd/CMV/V5-DEST-DNM2-WT or pAd/CMV/V5-DEST-DNM2-K44A construct. The recombinant adenoviruses harbouring the DNM2^WT^/DNM2^K44A^ gene were generated in HEK293 cells and titrated as described previously [21].

### Isolation of ventricular myocytes and adenoviral gene transfer

The protocol for animal experiments was approved by the Institutional Animal Care and Use Committee of Tongji University. Adult rat ventricular myocytes (ARVMs) were isolated from male Sprague-Dawley rats weighing 250–300 g, plated on laminin-coated dishes and cultured according to a protocol described previously [22]. Adenovirus-mediated gene transfer was performed by adding the vectors encoding DNM2^WT^ or DNM2^K44A^ into culture medium at the desired multiplication of infection (MOI). GFP- or β-gal-expressing viruses were used as a mock control. In some sets of the experiments, the infected cells were treated with zoniporide (Tocris, Minneapolis, MN, USA) at 1 μM for 36 hrs. The viability of the infected ARVMs was evaluated by Trypan Blue exclusion, as described elsewhere [23].

### Cell-surface biotinylation

The ARVMs were washed with cold PBS and incubated with 2 mg/ml sulfo-NHS-LC-Biotin (Pierce, Rockford, IL, USA) for 30 min. at 4°C. Unreacted biotin was quenched by a Quenching solution containing 100 mM glycine in PBS. The cells were subsequently lysed in RIPA buffer (150 mM NaCl, 50 mM Tris–HCl, pH 7.4, 1% sodium deoxycholate, 1% NP-40, 1 mM PMSF and 1 mM EDTA). The lysates containing biotinylated proteins were incubated with pre-washed streptavidin-agarose beads (Pierce) at 4°C overnight. After three washes, bound surface proteins were eluted and separated by SDS-PAGE.

### Co-immunoprecipitation and western blotting

The protein extracts from freshly isolated ARVMs were pre-cleaned by Protein G Sepharose (Sigma-Aldrich, St. Louis, MO, USA) and then incubated with 5 μg of primary antibodies for 2 hrs, followed by Protein G Sepharose for 1 hr. The Sepharose were washed five times and boiled in Laemmli buffer for analyses of the associated proteins by SDS–PAGE. Western blotting was performed with the primary antibodies against DNM2 (Abcam, Cambridge, MA, USA), NHE1 (Santa Cruz, Dallas, TX, USA), Bcl-2 (Beyotime, Jiangsu, China), Bax (Cell signaling, Boston, MA, USA), cleaved caspase-3 (Cell signaling, Boston, MA, USA) and GAPDH (Cell signaling, Boston, MA, USA).

### NHE1 trafficking experiments in HEK293 cells

NHE1 trafficking was studied in HEK293 cells, as reported previously [24]. The plasmid encoding human NHE1 and GFP (hNHE1-GFP) fusion protein was a gift from Dr. Chi-Hung Lin (National Yang-Ming University, Taiwan). One day before transfection, the cells were plated on coverslips in 35-mm dishes at 40–50% confluence. Transfection was performed with 1.6 μg of hNHE1-GFP plasmid and Lipofectamine 2000™ transfection reagent (Invitrogen) according to the manufacturer's instructions. After 24 hrs, Adv-DNM2^WT^, -DNM2^K44A^ or a mock vector was added for another 24 hrs prior to imaging analyses.

### Measurement of Na^+^/H^+^ exchanger activity

The activity of NHE1 was measured as the rate of Na^+^-dependent pH_i_ recovery following an acid load (NH_4_Cl pre-pulse) as described previously [25]. Briefly, cultured ARVMs were loaded with the membrane-permeable form of a pH_i_ indicator 2′, 7′-bis (carboxyethyl)-5 (6)-carboxyfluorescein ester (BCECF-AM) (1 μM, Dojindo Laboratories, Rockville, MD, USA) for 30 min. at 37°C. The cells were then rinsed with HEPES-buffered Tyrode's solution (in mM: NaCl 140, KCl 5, Hepes 10, CaCl_2_ 2, MgCl_2_ 1; pH 7.4 with NaOH). After the baseline pH_i_ was recorded, the cells were perfused with an acid-loaded solution based on the Tyrode's composition with the following substitutions: NaCl and KCl were, respectively, replaced with 20 mM NH_4_Cl and 120 mM *N*-methyl-D-glucamine (NMDG/Cl). Subsequently, normal Tyrode's solution was added to yield the NHE activity as the pH_i_ recovery rate. Throughout the experiment, the pH_i_ was monitored using a Leica SP5 inverted microscope. The cells were excited successively at 490 and 450 nm. The 490/450 emission ratio obtained from the intracellular BCECF-AM was converted to a linear pH scale using *in situ* data calibration, which was performed at the end of each experiment using the nigericin technique described elsewhere [26].

### TUNEL and Hoechst staining

*In situ* cell death was evaluated in the ARVMs with a terminal deoxynucleotidyl transferase–mediated dUTP nick-end labelling (TUNEL) assay kit (Roche Diagnostics, Indianapolis, IN, USA) according to the manufacturer's instructions. Hoechst 33258 (Beyotime) staining was subsequently performed on the same ARVMs. The percentage of the TUNEL-positive cells was determined by counting 200–300 cells over 10 randomly chosen fields (200×) in each coverslip.

### Measurement of caspase-3 activity

Caspase-3 activity was measured using a colorimetric assay kit (Beyotime) according to the manufacturer's instructions.

### Statistical analysis

Data are presented as means ± SEM. Statistical significance was determined by a one-way anova or unpaired Student's *t*-test where appropriate. A value of *P* < 0.05 was considered to be statistically significant.

## Results

### Sarcolemmal NHE1 trafficking is dependent on the DNM2 GTPase activity in the cardiomyocyte

**Fig. 1:**
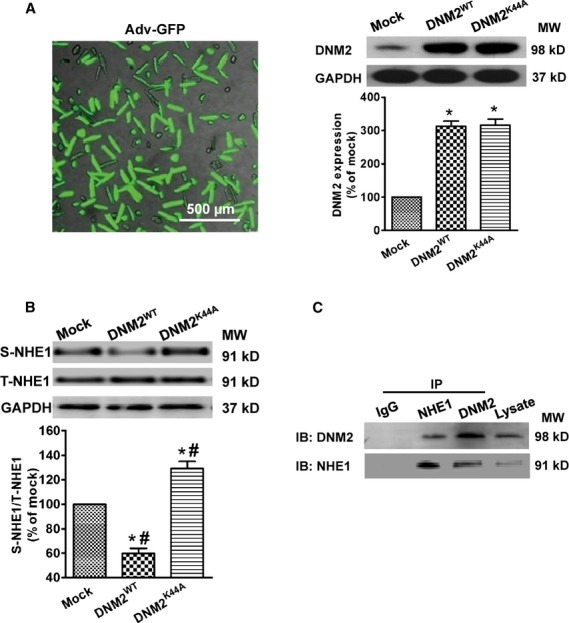
Sarcolemmal NHE1 trafficking is DNM2 activity dependent in ARVMs. (**A**) The transfection efficiency and overexpression of DNM2^WT^ or DNM2^K44A^ in ARVMs were detected by fluorescent microscopy and western blotting. *Left*: microscopic imaging of adenovirus-mediated GFP expression. *Right*: western bloting analysis of DNM2^WT^ and DNM2^K44A^ overexpression. GAPDH was used as a loading control. (**B**) Overexpression of DNM2^WT^ or DNM2^K44A^ induced a decrease or an increase in NHE1 protein expression in the cell surface respectively. A typical example of a western blot (upper panel) and the pooled data (lower panel) are shown. (**C**) A representative example of co-immunoprecipitation experiments for DNM2 and NHE1 proteins from freshly isolated ARVMs. Cell lysates were incubated with anti-NHE1, -DNM2 or -IgG (as a control) antibodies. The precipitates were then immunoblotted with the antibodies of DNM2 or NHE1. Data are expressed as means ± SEM of five independent experiments. *P < 0.05 compared with mock; #*P* < 0.05 compared with groups other than mock. S-NHE1: surface NHE1; T-NHE1: total NHE1; IB: immunoblot; IP: immunoprecipitation.

DNM2 is a ubiquitous DNM other than the neuron-specific DNM1 and DNM3 [15], and our previous study identified DNM2 as the single isoform of DNMs in the rat heart (J Li, L Xu, JC Ye, X Li, DS Zhang, DD Liang, XR Xu, M Qi, CM Li, H Zhang, J Wang, Y Liu, YH Chen, unpublished data). To determine whether the GTPase activity of DNM2 affects sarcolemmal NHE1 trafficking, we specifically enhanced or inhibited the DNM2 activity by overexpressing a WT DNM2 (DNM2^WT^) or a dominant-negative DNM2 mutant (DNM2^K44A^, defective for GTPase activity) in the cardiomyocytes respectively. The transfection efficiency was evaluated by fluorescent microscopy and western blotting at 24 hrs after infection. As shown in Figure 1A, the expression level of DNM2 was markedly up-regulated (three- to fourfold) by the overexpression of DNM2^WT^ or DNM2^K44A^. The expression of membrane-bound NHE1 was analysed by cell-surface biotinylation to detect the retrograde trafficking of NHE1 from the sarcolemma to the cytosol. Compared with the mock control, DNM2^WT^ overexpression reduced the surface NHE1 level, suggesting an enhanced internalization of sarcolemmal NHE1; whereas overexpression of DNM2^K44A^ led to an increase in NHE1 expression in the surface, indicating an impaired retrograde trafficking of NHE1 (Fig. 1B). We then investigated whether DNM2 regulated the NHE1 trafficking *via* a direct interaction. The protein extracts from freshly isolated ARVMs were immunoprecipitated with a specific antibody against NHE1 or DNM2. Immunoprecipitation with the anti-NHE1 antibody revealed the complex formation between NHE1 and DNM2. Similar results were obtained from the reciprocal experiments (Fig. 1C).

**Fig. 2:**
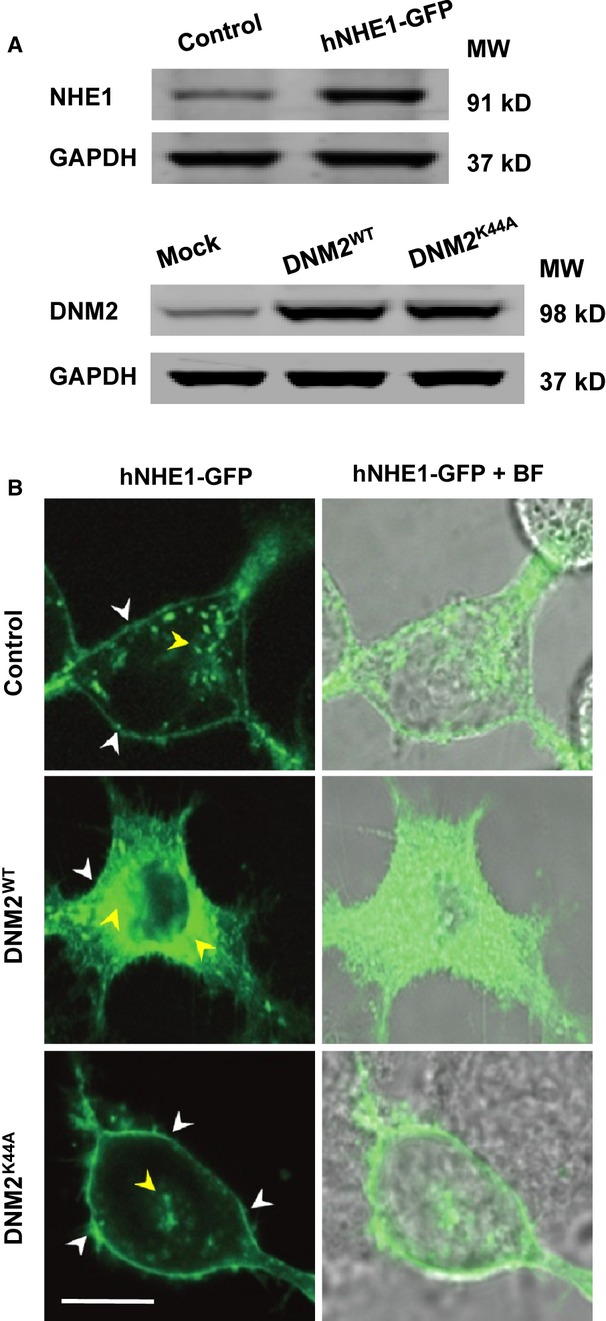
Verification of DNM2-dependent NHE1 trafficking in HEK293 cells. (**A**) The expression of exogenous NHE1 and overexpression of DNM2^WT^ or DNM2^K44A^ in HEK293 cells. (**B**) The confocal imaging of NHE1 distribution in transfected HEK293 cells. In mock cells that expressed NHE1 alone, the NHE1 was mainly located on the cell surface (white arrows) and only slightly distributed in the cytoplasm (as clusters demonstrated by yellow arrows). Overexpression of DNM2^WT^ induced the translocation of NHE1 from the cell surface to the cytoplasm, whereas DNM2^K44A^ overexpression impaired the retrograde trafficking of NHE1, resulting in the retention of NHE1 on the membrane surface. BF: bright field; bar indicates 10 μm.

The DNM2-dependent regulation of NHE1 trafficking was further tested in HEK293 cells. In the absence of exogenous DNM2^WT^ or DNM2^K44A^, NHE1 was mostly located on the cell surface with only a small fraction in the cytoplasm. Overexpression of DNM2^WT^ (Fig. 2A) caused a significant translocation of membrane NHE1 into the cytoplasm. In contrast, DNM2^K44A^ overexpression detained NHE1 protein on the cell surface (Fig. 2B).

### DNM2-dependent NHE1 trafficking is coupled with the change in NHE1 activity

**Fig. 3:**
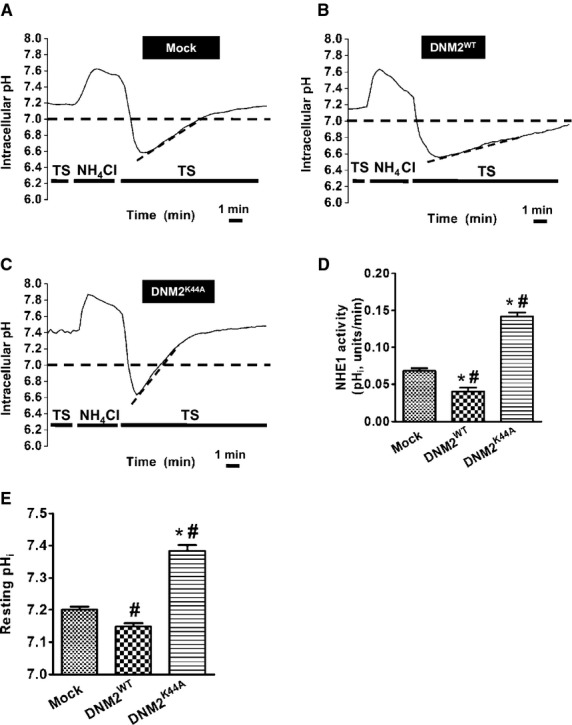
DNM2-dependent NHE1 trafficking is coupled to the change in NHE1 activity in ARVMs. (**A**–**C**) Representative pH_i_ traces of individual ventricular myocyte receiving the mock vector (**A**), DNM2^WT^ (**B**) or DNM2^K44A^ (**C**), showing a Na^+^-dependent pH_i_ recovery after exposure to NH_4_Cl. ARVMs were perfused with HEPES-buffered solution followed by 20 mM NH_4_Cl. The acute exposure to NH_4_Cl resulted in the acidification of the cytosol after removing NH_4_Cl. When the extracellular normal HEPES-buffered solution was reintroduced, the pH_i_ started to increase in response to the intracellular alkalinization, which occurred at a certain rate (dotted line) that reflected the NHE1 activity. TS: Tyrode's solution. (**D**) The activity of NHE1, indicated by the mean rates of the Na^+^-dependent pH_i_ recovery, differed among the cells receiving the mock, DNM2^WT^ or DNM2^K44A^ vector. (**E**) The effects of DNM2^WT^ or DNM2^K44A^ overexpression on the resting intracellular pH_i_. Data are expressed as means ± SEM of five independent experiments. *P < 0.05 compared with mock; #*P* < 0.05 compared with groups other than mock.

To determine whether the alteration in the DNM2-dependent NHE1 translocation modified NHE1 activity, we assayed the activity of NHE1 in the ARVMs overexpressing DNM2^WT^ or DNM2^K44A^. The response of these cells to an acute acid load induced by the application of NH_4_Cl in the extracellular solution was assessed. As shown in Figure 3A through C, when NH_4_Cl was removed and the Na^+^-Tyrode's solution was reintroduced, the pH_i_ began to recover because of the NHE1-mediated H^+^ extrusion. The rate of this Na^+^-dependent intracellular alkalinization, which was calculated from the point at which the pH_i_ started to recover and is indicated by the dotted line in each trace, represents the NHE1 activity. The Na^+^-dependent pH_i_ recovery was slower in the ARVMs overexpressing DNM2^WT^ than in the ARVMs receiving the mock vector (0.041 ± 0.001, *n* = 57 cells *versus* 0.068 ± 0.002 pH_i_ units/min., *n* = 50 cells), whereas a markedly accelerated pH_i_ recovery was observed in the ARVMs overexpressing DNM2^K44A^ (0.142 ± 0.001 pH_i_ units/min., *n* = 60 cells; Fig. 3D). In addition, compared with mock control or DNM2^WT^ overexpression, DNM2^K44A^ overexpression resulted in an elevated resting pH_i_ (Fig. 3E).

### Increased NHE1 activity induced by impaired retrograde trafficking contributes to apoptotic death in DNM2-defective ARVMs

**Fig. 4:**
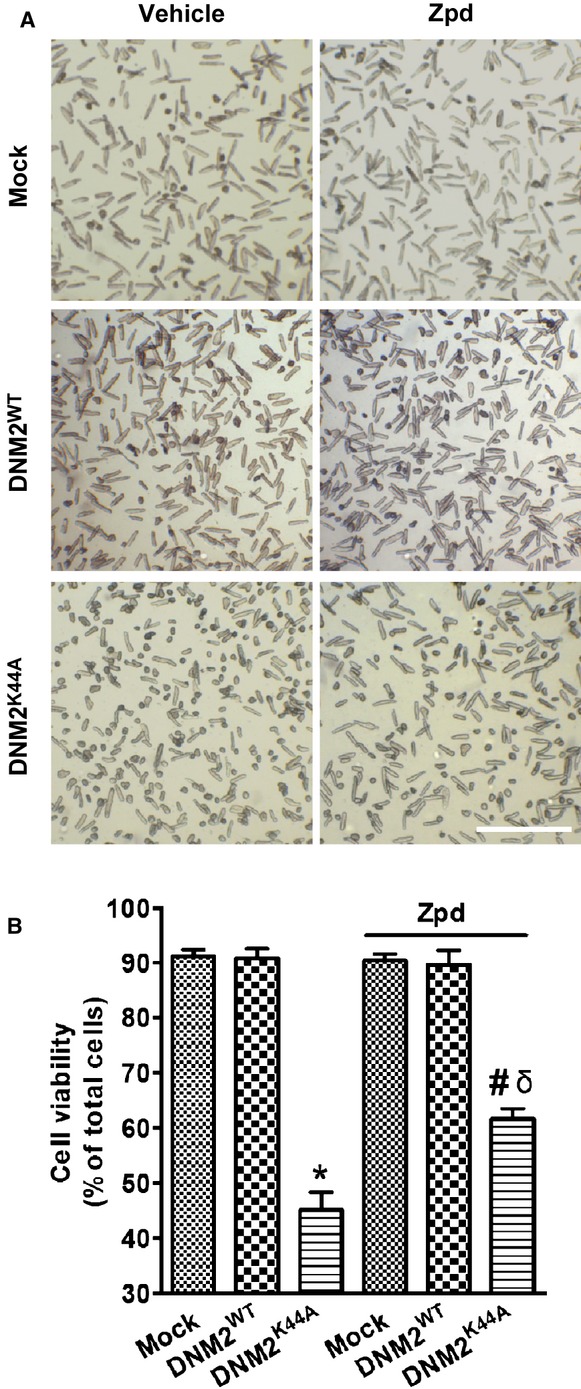
The enhanced activation of NHE1 induced by DNM2 deficiency reduces cell viability in ARVMs. Cell viability was measured by staining with trypan blue to confirm cell membrane integrity. (**A**) Representative micrographs of the ARVMs infected with the mock, DNM2^WT^, or DNM2^K44A^ vector at 36 hrs in the presence or absence of the NHE1 inhibitor zoniporide. (**B**) Data collection from A. Similar results were observed in five independent experiments. *P < 0.05 compared with mock; #*P* < 0.05 compared with DNM2^K44A^; δ*P* < 0.05 compared with mock plus zpd. Zpd: zoniporide; bar indicates 500 μm.

We next explored the potential effect of the DNM2 deficiency–induced NHE1 activation on the viability of ARVMs. As shown in Figure 4, the viability of the DNM2^K44A^-ARVMs (45.2 ± 1.3%, *n* = 5) was markedly lower than that in mock control (92.5 ± 1.6%, *n* = 5) or the DNM2^WT^-ARVMs (91.8 ± 1.4%, *n* = 5). The treatment with zoniporide, a selective NHE1 inhibitor [27], improved the viability of DNM2^K44A^-ARVMs (61.6 ± 1.8%, *n* = 5), and had no effect on the cell viability in either the DNM2^WT^-ARVMs or the mock control, suggesting the involvement of NHE1 activation in the ARVM death.

**Fig. 5:**
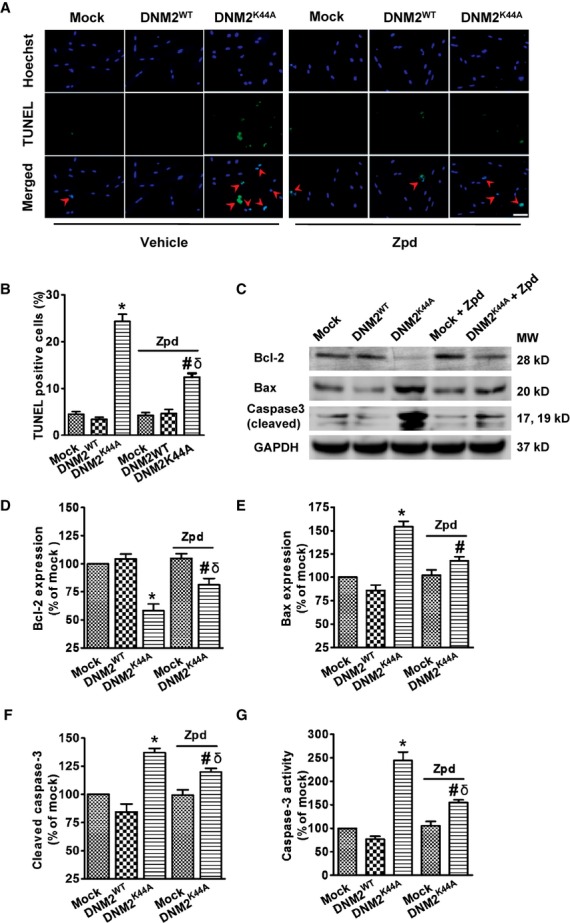
NHE1 activation induced by trafficking impairment induces apoptotic death in DNM2-defective ARVMs. (**A**) Representative images of ARVMs transfected with mock vector, DNM2^WT^ and DNM2^K44A^ in the presence or absence of zoniporide. Apoptotic cells were visualized by fluorescence microscopy using the TUNEL method (green), Hoechst33258 staining of nuclei (blue) and merged images (cyan blue) showing DNA fragmentation and the condensation of nuclei (red arrow). (**B**) Pooled data from A. (**C**) A typical western bloting analysis showing the expression levels of Bcl-2, Bax and cleaved caspase-3 in transfected ARVMs. GAPDH was used as a loading control. (**D**–**F**) Data collection from C. (**G**) Caspase-3 activity in transfected ARVMs. Data are presented as means ± SEM of five independent experiments. *P < 0.05 compared with mock; #*P* < 0.05 compared with DNM2^K44A^; δ*P* < 0.05 compared with mock plus zpd. Zpd: zoniporide; bar indicates 50 μm.

Hoechst33258 staining and TUNEL assay were used to determine the contribution of apoptosis to the loss of cell viability in DNM2^K44A^-ARVMs. The percentage of TUNEL-positive nuclei was greater in the ARVMs infected with Adv-DNM2^K44A^ (23.2 ± 2.1%, *n* = 5) than in the ARVMs infected with the mock vector or Adv-DNM2^WT^ (mock: 4.3 ± 0.6%, *n* = 5; DNM2^WT^: 3.4 ± 0.6%, *n* = 5). The application of zoniporide reduced the percentage of TUNEL-positive cells in the DNM2^K44A^-ARVMs (12.5 ± 0.8%, *n* = 5; Fig. 5A and B). These results implicated that sarcolemmal NHE1 activation induced by the defective DNM2-dependent trafficking contributes to the apoptotic death of ARVMs.

The DNM2- and NHE1-associated apoptosis in ARVMs was further substantiated by the finding that the DNM2^K44A^ overexpression reduced the level of Bcl-2 protein, which functions at the contact sites of the mitochondrial membrane to inhibit apoptosis [28]. Moreover, the level of pro-apoptotic Bax was increased by the overexpression of DNM2^K44A^. When zoniporide was applied, the alteration in Bcl-2 or Bax level was significantly alleviated (Fig. 5C–E).

Caspase-3 activation is the terminal step in the apoptotic pathway [29]. Using western blotting and caspase activity measurements, we detected the endogenous levels of the large fragment of activated caspase-3 and the caspase-3 protease activity. As shown in Figure 5C, F and G, overexpression of DNM2^K44A^ but not DNM2^WT^ induced an increase in caspase-3 activity, which was partially inhibited by zoniporide.

## Discussion

In this study, we demonstrate for the first time that in ARVMs, sarcolemmal NHE1 activity is regulated by a DNM2-dependent trafficking process. DNM2 up-regulation decreased the NHE1-mediated H^+^ extrusion, whereas its deficiency led to an augment in the NHE1 activity. Interestingly, NHE1 activation elicited by DNM2 deficiency induced cardiomyocyte apoptosis, which can be partially rescued by pharmacological inhibition of NHE1 activity.

NHE1 has been primarily considered to be a membrane ‘housekeeping’ resident protein, yet the regulation of sarcolemmal NHE1 translocation in cardiomyocytes has not been explored. Recent studies indicate that membrane NHE1 has a half-life of ∼24 hrs [30] and that increased metabolic activity or ischemia can modify the translocation of NHE1 [31], which suggests the existence of dynamic trafficking of membrane NHE1. In this study, we have investigated whether a net translocation of NHE1 to the sarcolemma of cardiomyocytes was regulated by DNM2, a key player in intracellular internalization, and we confirmed the occurrence of this process through cell-surface biotinylation and complementary techniques of immunofluorescence microscopy. Our results indicate that the membrane expression of NHE1 was changed in response to the alteration of DNM2 activity (Fig. 1B), and this finding was corroborated in HEK293 cells overexpressing exogenous NHE1 (Fig. 2). Moreover, co-immunoprecipitation revealed a tight association between NHE1 and DNM2 in ARVMs (Fig. 1C). Taken together, these data suggest that the trafficking of sarcolemmal NHE1 between cell surface and cytoplasm can be regulated by DNM2 activity.

Internalization of plasmalemmal transporters is not only involved in their catabolism but also in the regulation of the number of the membrane-bound transporters, thereby affecting their activity [32]. The activation of intrinsic catalytic activity of NHE1 has been extensively studied and established experimentally [33]. We hereby report a DNM2-mediated endocytic regulation of sarcolemmal NHE1 activity in cardiomyocytes. The confocal microscopy of the ARVMs loaded with BCECF-AM showed that the NHE1 activity was decreased in the DNM2^WT^-overexpressing cells, although it was raised by the overexpression of DNM2^K44A^, accompanied by the upregulation of resting pH_i_ (Fig. 3). The increase in the surface amount of NHE1 as a result impaired endocytosis may contribute to the activation of NHE1 and lead to enhanced H^+^ extrusion, resulting in the intracellular alkalinization. In contrast, the reduction in surface NHE1 expression caused by excessive internalization decreased the activity of NHE1. The relatively small change in the NHE1 activity and the lack of a significant drop of the resting pH_i_ in the DNM2^WT^-overexpressing cells was probably attributed to the minimal activity of sarcolemmal NHE1 under basal condition [3] and/or the involvement of mitochondrial NHE activity [34].

The activation of NHE1 has been convincingly demonstrated to regulate cardiomyocyte apoptosis and to be deleterious in a number of pathophysiological conditions, including left ventricular hypertrophy, congestive heart failure and myocardial infarction [35, 36]. This study demonstrates that the deficiency of DNM2 leads to a decreased cell viability and promotes apoptotic cell death, which is partially reversed by the inhibition of NHE1 activity (Figs 4, 5A and B), suggesting an important role for NHE1 activation in the regulation of cardiomyocyte apoptosis. Several lines of evidence support the deleterious effects of the enhanced NHE1 activation in the DNM2 deficiency–mediated cardiomyocyte death. On one hand, excessive Na^+^ influx through the activated NHE1 may activate the sarcolemmal Na^+^/Ca^2+^ exchanger, leading to the augmentation of Ca^2+^ influx into the cytosol and the ensuing cell death [37, 38]. On the other hand, the intracellular alkalinization by the overactivation of NHE1 may be an early event in the apoptotic cascade [39], occurring prior to caspase activation and DNA fragmentation. Moreover, a reduced expression of DNM2 and an activation of NHE1 were also found in ischaemic myocardium of rat (data not shown), further underscoring the potential pathological importance of DNM2 deficiency–mediated NHE1 activation in the heart.

Caspase-3 represents the common pathway of the caspase cascade and has been implicated in the apoptosis of cardiomyocytes [40]. The results that the caspase-3 activity was elevated by the overexpression of DNM2^K44A^ (Fig. 5C, F and G) confirmed the DNM2 deficiency–mediated apoptosis. Bax contributes to the progression of apoptosis by disrupting the function of mitochondrial membranes, whereas Bcl-2 binds to and inactivates Bax to prevent apoptosis [41]. A robust increase in Bax expression and a reduction in Bcl-2 level were observed in the DNM2-defective ARVMs (Fig. 5C–E), suggesting the activation of the mitochondrial-dependent apoptosis pathway. The use of zoniporide partially alleviated the alteration in the expression levels of Bcl-2, Bax and cleaved caspase-3, underlying the involvement of NHE1 activation. The lack of full rescue from apoptosis by zoniporide suggests that there may be targets other than NHE1 that can be regulated by DNM2 and also contribute to the maintenance of cell survival. These issues remains to be addressed in future studies.

In summary, this study demonstrates that the activity of sarcolemmal NHE1 can be regulated by a DNM2-dependent trafficking. The enhanced activation of NHE1 induced by DNM2 deficiency leads to cardiomyocyte apoptosis. These findings provide a novel mechanism by which the aberrant NHE1 trafficking affects cardiomyocyte survival and may offer new insights regarding the pathogenesis and management of myocardial injury.
